# Supplementary material for: Therapeutic potential of macrophage colony-stimulating factor in chronic liver disease
Source: Dis Model Mech. 2022 Apr 19;15(4):dmm049387. doi: 10.1242/dmm.049387 (PMC9044210; doi:10.1242/dmm.049387)
Supplement: Supplementary information [file dmm-15-049387-s1.pdf]

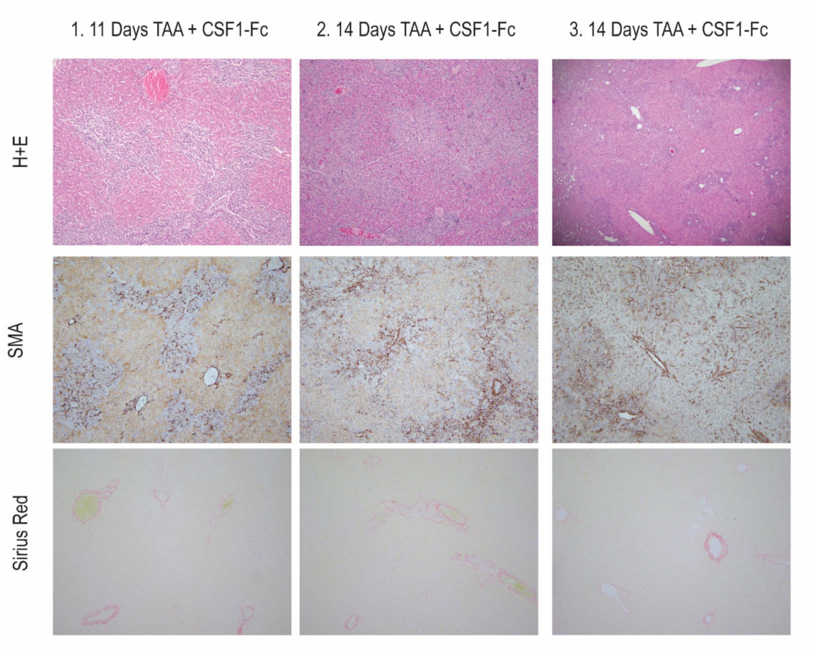

Fig. S1. Co-administration of CSF1-Fc and TAA exacerbated liver inflammation. Female C57/B16 mice were administered 300 mg/L TAA in drinking water in conjunction with bi-weekly treatment with 1 mg/kg pCSF1-Fc commencing on Day 1. Mice 1 and 2 died unexpectedly 11 and 14 days post commencement and mouse 3 was culled on day 14. Liver sections were stained with H+E, anti-SMA antibody or picrosirius red. 10 x magnification.

Disease Models & Mechanisms • Supplementary information

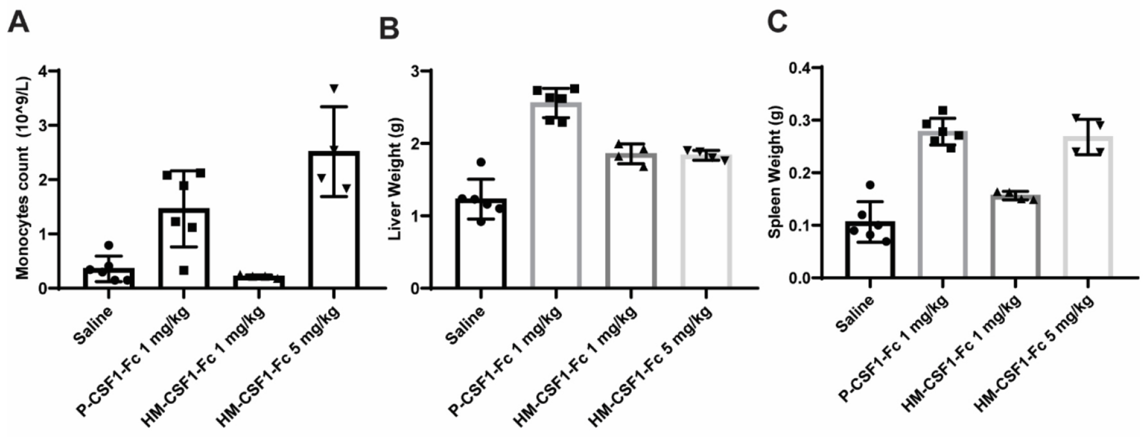

Fig. S2. Comparison of biological impacts of P-CSF-Fc and HM-CSF1-Fc. Male C57/Bl6 mice were administered 4 successive daily injections of porcine (P)-CSF1-Fc or a human CSF1-mouse Fe conjugate (HM-CSF1-Fc). (A) circulating monocyte count, (B) liver weight and (C) spleen weight at sacrifice.

Disease Models & Mechanisms • Supplementary information

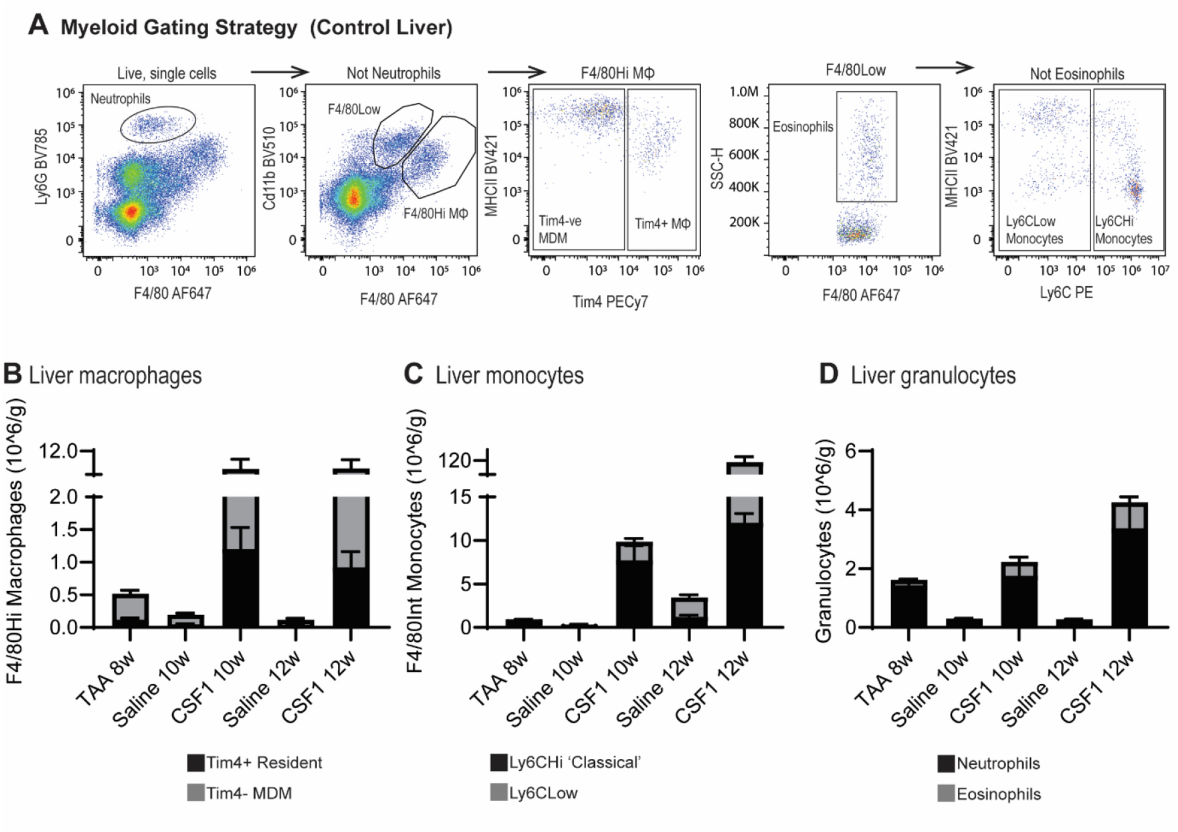

Fig. S3. CSF1-Fc treatment increases the abundance of hepatic classical and non-classical monocytes, monocyte-derived macrophages and kupffer cells during regression of TAA-induced fibrosis. Groups of 6 female mice were treated with TAA for 8 weeks, followed by bi-weekly treatment with 1 mg/kg P-CSF1-Fc for up to 4 weeks. Hepatic non-parenchymal cells were isolated from disaggregated livers for flow cytometry analysis at baseline (TAA 8w) or following 2 or 4 weeks CSF1-Fc treatment (CSF110w and 12w, respectively. (A) Myeloid cell ating strategy. Number of (B) nm4+ and Tim4-F4/80Hi macrophages, (C) Ly6CHi and Ly6Clow F4/801nt monocytes, (D) Ly6G+ neutrophils and F4/801nt/SSCHi eosinophils per gram of liver.

Disease Models & Mechanisms • Supplementary information

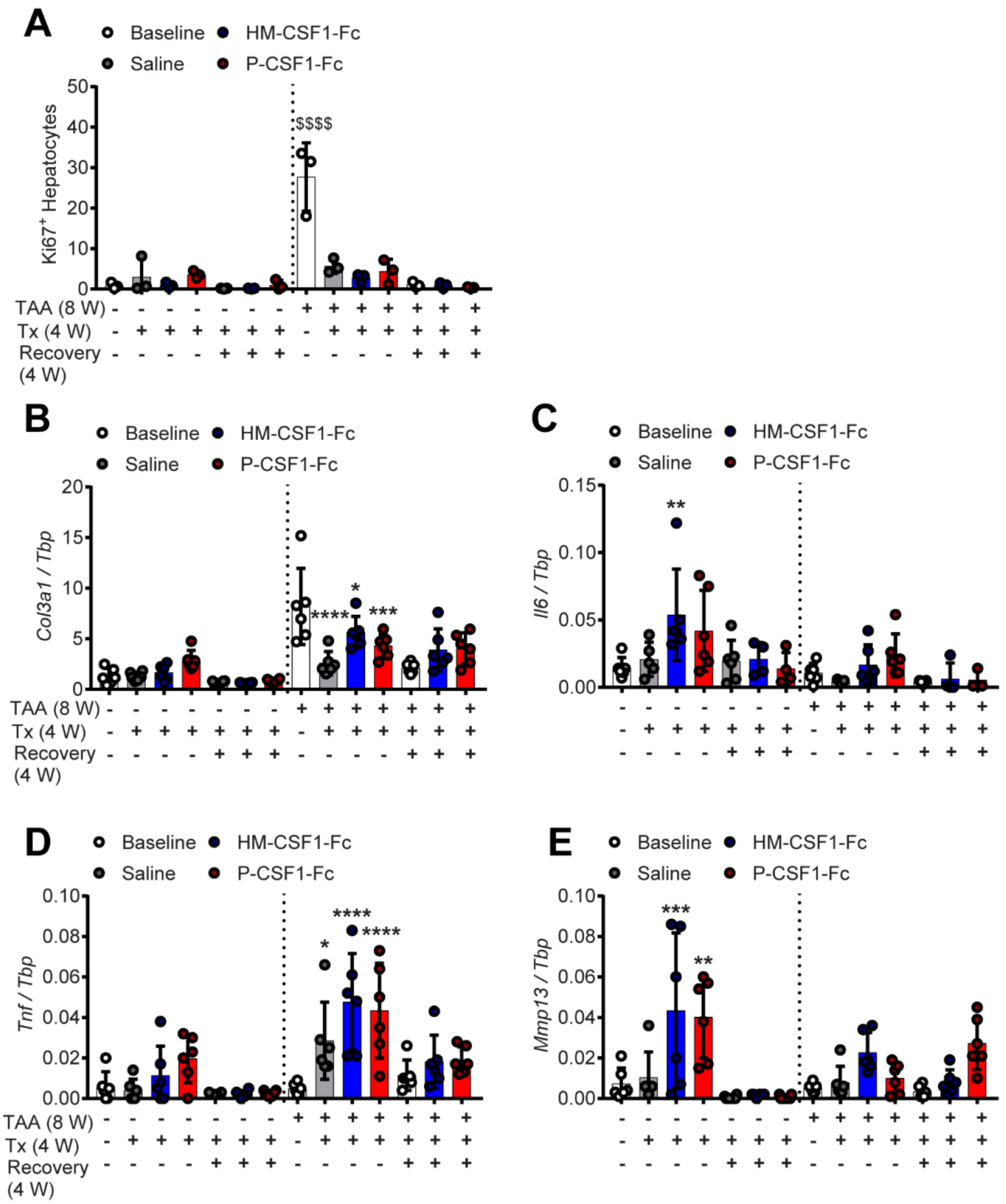

Fig. S4. Impacts of chronic CSFI-Fc treatment on hepatic gene expression. Experimental design as in Figure 1A. (A) Quantification of Ki67+ hepato-cytes by image analysis of IHC staining. Expression of Col3a 1 (B), 116 (C), Tnf (D), and Mmpl 3 (E) in whole liver was quantified by RT-PCR. One-Way ANOVA with multiple comparison, *p<0.05, **p<0.01, ***p<0.001, ****p<0.0001 comparing to baseline in same group.

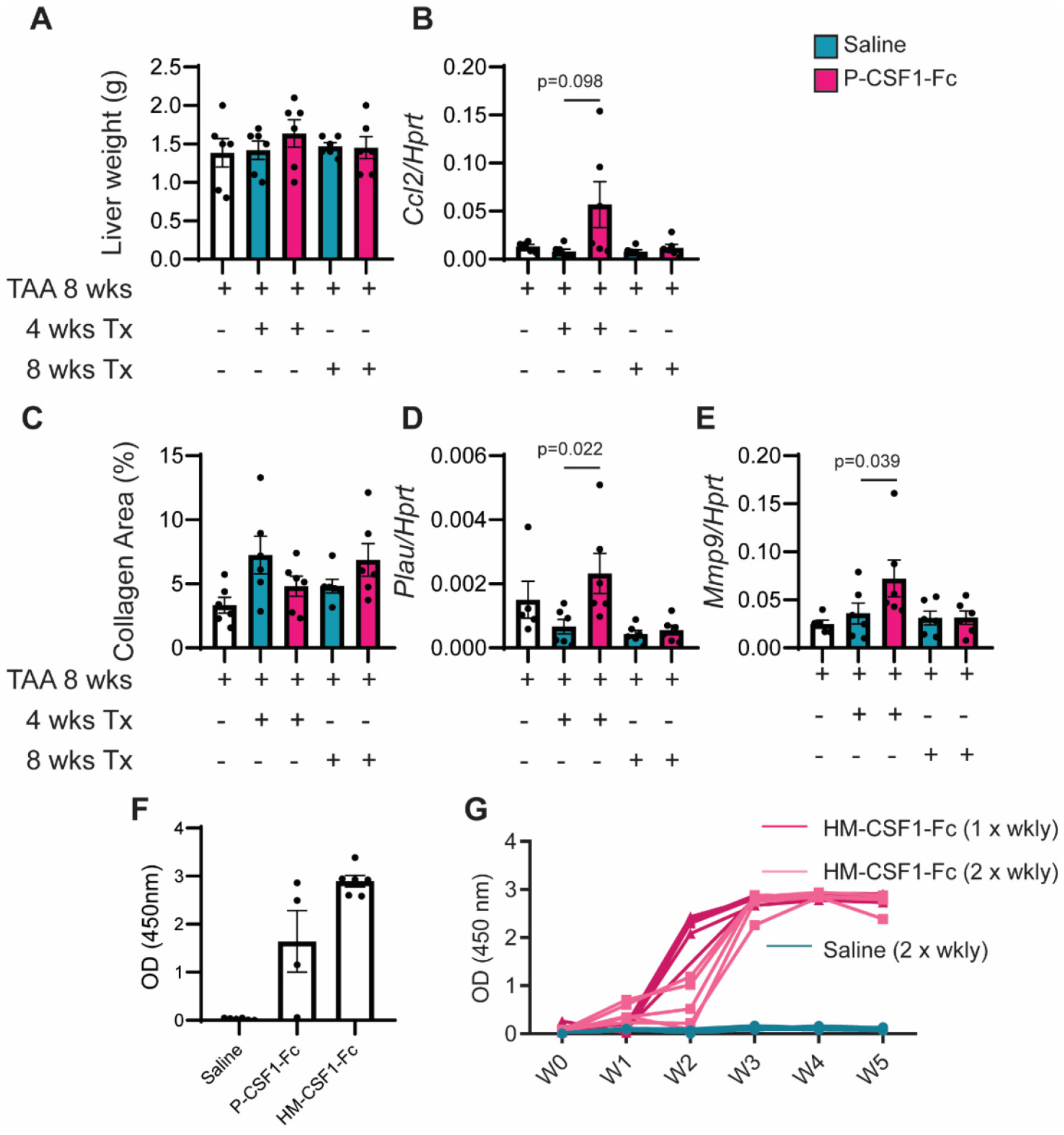

Fig. S5. Transient response to extended CSF1-Fc treatment is associated with anti-CSF1-Fc antibodies. Groups of 6 female C57/B16J mice were administered TAA in drinking water for 8 weeks, then returned to normal drinking water and treated with 1 mg/kg P-CSF1-Fc or saline control once weekly for 8 weeks. (A) Liver weight at sacrifice. Liver Ccl2 (b) and Adgre1 (?) mRNA expression. Liver fibrosis (collagen area) (C). Liver Plau (D) and Mmp9 (E) expression. Kruskal-Wallis, Dunn's multiple comparison test. (F) Mice were treated once weekly with P-CSF1-Fc or HM-CSF1-Fc for 4 weeks and serum antibodies to the respective CSF1-Fc reagent were detected by ELISA (1:1000 serum dilution). (G) Mice were treated once or twice weekly with HM-CSF1-Fc with weekly blood collection. Anti-CSF1-Fc antibodies in serum (1:1000 dilution) were detected by ELISA.
